# The screening of diagnostic biomarker microRNA in first-trimester maternal plasma for Down syndrome: A study protocol

**DOI:** 10.1097/MD.0000000000028495

**Published:** 2022-01-21

**Authors:** Fengping He, Xiangkang Yang

**Affiliations:** Department of Prenatal Diagnosis Laboratory, Zhoushan Women and Children's Hospital, Zhoushan, Zhejiang, China.

**Keywords:** biomarker, diagnostic, Down syndrome, microRNA

## Abstract

**Background:**

The trisomy of human chromosome 21 causes Down syndrome (DS), sometimes known as congenital folly's syndrome. The survivors show apparent mental impairment, unusual facial traits, growth and development abnormalities, and various deformities, with 60 percent of the infants having miscarriages in the early stages of the fetus. Plasma micRNA (miRNA) is a new diagnostic biomarker for DS; however, its significance in first-trimester maternal plasma is unknown. As a result, the purpose of this study is to assess the diagnostic significance of the biomarker miRNA in first-trimester maternal plasma for DS.

**Materials and methods:**

From January 2014 until the present, blood samples were obtained from pregnant women who visited our hospital. This study included 20 eligible DS pregnancies and 20 normal pregnant women. We looked at the differential miRNA expression profile in DS maternal plasma from the first and second trimesters using miRNA microarrays. Bioinformatics technology was used to compare the particular miRNA in DS maternal plasma from the first and second trimesters and screen the miRNA co-expressed in DS maternal plasma. Meanwhile, the expression level of chosen miRNAs was verified using quantitative real-time PCR (qRT-PCR).

**Discussion:**

This study aims to see how useful the diagnostic biomarker miRNA in first-trimester maternal plasma is for diagnosing DS. The findings of this investigation will provide clinical evidence for the discovery of a new diagnostic biomarker miRNA in first-trimester maternal plasma for DS diagnosis.

**OSF regression number:**

DOI 10.17605/OSF.IO/R49FT.

## 1 Introduction

Down syndrome (DS), sometimes known as congenital folly's syndrome, is the most prevalent chromosomal abnormality linked to intellectual impairment. It is also marked by several other clinical symptoms, such as congenital heart disease, respiratory insufficiency, and gastrointestinal problems.^[1–3]^ It occurs in around 1 out of every 800 births across the world, with modest variations in the frequency and presentation of DS depending on ethnic background and geographic area, and it rises with the pregnant woman's age.^[1,4]^ Although disparities in access to health care and other supporting resources still persist, the potential for the development and socialization of people with DS is increasingly recognized, and early assistance for affected children and their families is extensively adopted. There is much phenotypic diversity among patients, and intellectual disability is usually moderate but can vary from mild to severe, but social function is frequently good compared to cognitive impairment.

Alpha-fetoprotein (AFP), human chorionic gonadotropin (hCG and its subunits), unconjugated estriol (uE3), a disintegrin and metalloproteinase-12, pregnancy-associated plasma protein A, and inhibin A are some of the maternal serum biochemical markers which have been used to screen for DS in the first and second trimesters of maternal.^[5–8]^ The risk of DS pregnancy was assessed using 3 biochemical markers, including double markers (pregnancy-associated plasma protein A and β-hCG), triple markers (AFP, hCG, and uE3), and quad markers (AFP, hCG, uE3, and inhibin A), in combination with the age and weight of pregnant women, gestational age, and other factors, in the Maternal Serum Screening Test.^[9]^ As a result, despite the fact that this screening mode is noninvasive prenatal screening, which pregnant women generally embrace and support, the false-positive rate is rather high, requiring some healthy pregnant women to undertake invasive diagnostic measures at the risk of miscarriage. As a result, innovative, extremely sensitive, specific, noninvasive, and robust screening approaches for early clinical detection of DS in first-trimester maternal plasma are urgently needed.

## 2 Materials and methods

### 2.1 The design for the study

This study aims to assess the use of the diagnostic biomarker miRNA in first-trimester maternal plasma for DS. Blood samples were taken from pregnant women who visited our hospital between January 2014 to date. A total of 40 patients will be enrolled from the Zhoushan Women and Children's Hospital's prenatal diagnostic department. The current study will be developed using the 2013 Standard Protocol Items as a guide.^[10]^

### 2.2 Ethics and registration

This procedure complies with the Helsinki Declaration and has been approved by our hospital's Ethics Committee. The Open Science Framework has been used to register this research (OSF, registration number: 10.17605/OSF.IO/R49FT). All patients will provide written informed consent prior to the trial.

### 2.3 Participants

Preeclampsia, pregnancy-induced hypertension, and hyperemesis gravidarum. All volunteers and subjects were subjected to the DS maternal selection criteria and exclusion criteria to determine the subjects. Samples were chosen from the existing cohorts based on the following inclusion and exclusion criteria: Inclusion criteria: expectant moms who met the diagnostic criteria, were between the ages of 18 and 35, and consented to be part of the study and completed an consent form; Patients with pregnancy-related problems, such as pregnancy-induced hypertension, are excluded. The pregnant women's peripheral blood was obtained during the first trimester (10-14 weeks) and the second trimester (15-20 weeks). Blood samples were obtained into EDTA tubes and centrifuged at 3000 × *g* for 10 minutes. The upper part of the plasma was removed and kept for cell culture. All samples were snap-frozen in liquid nitrogen and kept at −80 °C until extraction, and a clinical sample database was created for each sample (including 20 patients from pregnant women in the first- and second-trimester maternal).

### 2.4 Differential miRNA expression profile in the first- and second-trimester maternal plasma

Frozen cells were promptly thawed in a 37 °C water bath after being withdrawn from liquid nitrogen. Agilent Technologies’ miRNA arrays were utilized to determine the amount and content of miRNA. The RNA samples were labelled and processed in accordance with the manufacturer's instructions. In a nutshell, calf intestinal alkaline phosphatase was used to dephosphorylate 100 ng of total RNA, which was then denaturated with heat in the presence of dimethyl sulfoxide. T4RNA ligase then attached a cyanine dye, cyanine3-cytidine bisphosphate (pCp), to the dephosphorylated single-stranded RNA (including miRNA). MicroBioSpin 6 segments (Bio-Rad) were used to eliminate any unincorporated cyanine dye from the samples. The purified labelled miRNA probes were hybridized to 8 15 K human miRNA microarrays in a rotating hybridization oven at 20 rpm for 20 hours at 55 °C. The arrays were washed in Agilent gene expression Wash Buffer 1 with Triton X-102 after hybridization, and then in Agilent GE Wash Buffer 2 with Triton X-102. The slides were scanned at 5 μm resolution. Agilent's Feature Extraction software version-10.7 (Santa Clara, CA, USA) was used to quantify the obtained pictures. Using the Agilent miRNA gene arrays methodology, differentially expressed miRNAs were discovered.

The reversal of the transcribe RNA samples into cDNA were carried out through the TransScript One Step gDNA Removal and cDNA Synthesis SuperMix. TB Green Premix Ex Taq was used for RT qPCR.

An underlying advance of 95 °C for 10 minutes was trailed by 40 patterns of enhancement and measurement (95 °C for 15 seconds, 60 °C for 1 moment, and 60 °C for 1 moment). For standardization, GAPDH was used as an endogenous control.

### 2.5 Statistical analysis

The statistical significance was maintained at *P* < .05. The significance of a relationship between groups or proportions was determined using the Kruskal-Wallis *χ*^2^ test. For the analysis, SPSS of version 25.0 (IBM SPSS 24.0, SPSS Inc.) was used. The means of data were compared using the Student *t* test.

## 3 Discussion

Intellectual incapacity is one of the symptoms of DS a common chromosomal disorder. It is accompanied by other symptoms such congenital heart disease, respiratory failure, and gastrointestinal problems.^[1–3]^ This is a retrospective study to see if the diagnostic biomarker miRNA can be used to detect DS in first-trimester maternal plasma. We also expect this research to pave the way for a novel diagnostic tool for DS in first-trimester maternal plasma.

## Author contributions

**Conceptualization:** Fengping He, Xiangkang Yang.

**Data curation:** Fengping He, Xiangkang Yang.

**Formal analysis:** Fengping He, Xiangkang Yang.

**Funding acquisition:** Xiangkang Yang.

**Investigation:** Fengping He, Xiangkang Yang.

**Methodology:** Fengping He.

**Project administration:** Xiangkang Yang.

**Resources:** Fengping He, Xiangkang Yang.

**Software:** Fengping He.

**Supervision:** Xiangkang Yang.

**Validation:** Fengping He.

**Visualization:** Fengping He, Xiangkang Yang.

**Writing – original draft:** Fengping He.

**Writing – review & editing:** Xiangkang Yang.
